# Nodulation Characterization and Proteomic Profiling of *Bradyrhizobium liaoningense* CCBAU05525 in Response to Water-Soluble Humic Materials

**DOI:** 10.1038/srep10836

**Published:** 2015-06-08

**Authors:** Tong Guo Gao, Yuan Yuan Xu, Feng Jiang, Bao Zhen Li, Jin Shui Yang, En Tao Wang, Hong Li Yuan

**Affiliations:** 1 State Key Laboratory of AgroBiotechnology and MOA Key Laboratory of Soil Microbiology, College of Biological Sciences, China Agricultural University, Beijing 100193, P. R. China; 2 College of Life Science, Agricultural University of Hebei, Baoding 071001, P. R. China; 3 Escuela Nacional de Ciencias Biológicas, Instituto Politécnico Nacional, México D.F. 11340, Mexico

## Abstract

The lignite biodegradation procedure to produce water-soluble humic materials (WSHM) with a *Penicillium* stain was established by previous studies in our laboratory. This study researched the effects of WSHM on the growth of *Bradyrhizobium liaoningense* CCBAU05525 and its nodulation on soybean. Results showed that WSHM enhanced the cell density of CCBAU05525 in culture, and increased the nodule number, nodule fresh weight and nitrogenase activity of the inoculated soybean plants. Then the chemical compounds of WSHM were analyzed and flavonoid analogues were identified in WSHM through tetramethyl ammonium hydroxide (TMAH)-py-GC/MS analysis. Protein expression profiles and *nod* gene expression of CCBAU05525 in response to WSHM or genistein were compared to illustrate the working mechanism of WSHM. The differently expressed proteins in response to WSHM were involved in nitrogen and carbon metabolism, nucleic acid metabolism, signaling, energy production and some transmembrane transports. WSHM was found more effective than genistein in inducing the *nod* gene expression. These results demonstrated that WSHM stimulated cell metabolism and nutrient transport, which resulted in increased cell density of CCBAU05525 and prepared the bacteria for better bacteroid development. Furthermore, WSHM had similar but superior functions to flavone in inducing *nod* gene and nitrogen fixation related proteins expression in CCBAU05525.

Soybean is an important legume that forms symbiotic root nodules with *Bradyrhizobium liaoningense*, bacteria that reduce atmospheric nitrogen to ammonium, which is a form of nitrogen available for plant uptake. To initiate this symbiosis process, flavonoids secreted by soybean would activate the transcription of *nod* genes of rhizobia, which encode lipochitooligosaccharide signal molecules, also termed Nod factors. After being recognized by the epidermal cells of the host, these Nod factors induce root hair curling and cell division, and result in root nodule formation. Rhizobia entrapped in the curled root hair could enter the roots through infection threads^1^.

Agricultural systems could acquire approximately 40 million tonnes of nitrogen each year through symbiotic nitrogen fixation between legumes and rhizobia^2^. Symbiotic nitrogen fixation is a gift from the nature which is beneficial to increasing crop yields and nutrient-use efficiency meanwhile reducing N fertilizer application. Thus, practices to stimulate nodulation, such as inoculation of soybean with rhizobia, are of great significance to sustainable agriculture; and great efforts have been made to improve the efficiency of symbiotic nitrogen fixation. Previously, the application of whey (40 tonne ha^−1^) and inoculation with rhizobia enhanced the nodule numbers, crop growth and yield of chickpea under field conditions, which was speculated that these might be resulted from the positive effects of organic and inorganic nutrients in the whey on the plant development and the stimulation effects of whey on rhizobia activity^3^. Supplement of pea seeds with Nod factors secreted by *Rhizobium leguminosarum* bv. viciae strain GR09 before planting resulted in a significant increase in nodule numbers, nitrogenase activity and pea yield under greenhouse and field conditions^4^,^5^. Treatment with flavonoids such as luteolin or increased flavonoids production in the plant through genetic engineering enhanced both the number of nodules and N_2_ fixation of alfalfa^6^. Flavonoids were the most potent compounds for inducing *nod* gene expression, while other non-flavonoid compounds such as betaines and xanthones were also able to induce *nod* genes in rhizobia. However, these molecules could not move in the soil freely because of their positive charges^7^,^8^. Furthermore, the functioning mechanism of whey was unclear, and it was economically infeasible to apply these compounds in the field to increase legume yield. Thus, it is of value to search for other compounds that could enhance the symbiotic nitrogen fixation efficiency and be applicable under field conditions to improve legume yield.

Humic substances are polyelectrolytic macromolecular compounds originated from the chemical and biological degradation of plants and animal residues and microbial cells^9^, and they play an important role in the global carbon and nitrogen cycling. Humic materials were reported to have some positive effects on different organisms, specifically through accelerating seed germination^10^, improving rhizome growth by stimulating ATPase activity or functioning as auxin-like phytohormone to promote the plant growth^11^,^12^, and promoting the efficient utilization of nutrients by plants^13^,^14^. However, the effects of humic materials on symbiotic nitrogen fixation had been rarely investigated except in the reports of Tan and Tantiwiramanond^15^ that soil humic and fulvic acids extracted by 0.1 M NaOH could increase the plant dry weight and nodule mass but not the nodule number of soybeans and peanuts under greenhouse conditions. In addition, Til’ba and Sinegovskaya^16^ proved in a field experiment that seed coating with sodium humate, rhizobia, ammonium molybdate and leaf spraying with sodium humate increased the nodule number and nitrogen-fixing efficiency of soybean, and the yield hence increased by 22% compared with the control. Even though, the exact role of humic materials in symbiotic nitrogen fixation and its mechanism were still unclear. Recently, the production of water-soluble humic acids (WSHM) by the biodegradation of lignite using a fungal strain *Penicillium* sp. P6 or an alkali-producing bacterial community was reported^17^,^18^. Furthermore, it was found previously that these humic acids could protect against the disruption of bacterial diversity that ensued from the application of urea to the soil^19^, and the soybean productivity was increased by 12.6% ~ 26.3% when this extract of humic acids was applied in fields (our unpublished data). In order to investigate the effects of WSHM on the growth and nodulation of rhizobia, we performed this study using *Bradyrhizobium liaoningense* CCBAU05525 as a model, and the functioning mechanisms of WSHM were estimated through proteomics for the first time.

## Results

### The effects of WSHM on nodulation of soybean under greenhouse conditions

The symbiotic features of soybean inoculated with *B. liaoningense* CCBAU05525 in responding to the supplement of WSHM under greenhouse conditions were shown in Table 1. The number of nodules per plant increased by 19.4% and 30.5% respectively in the 300 and 500 mg L^−1^ WSHM treatments, but no significant increase was observed in the 1000 mg L^−1^ WSHM treatment (Table 1). Moreover, the nodule fresh weight in the 500 mg L^−1^ WSHM treatment increased by 36.0% compared with control. Furthermore, the nitrogenase activity per plant significantly increased (from 15.1% to 30.2%) in the 300, 500 and 1000 mg L^−1^ WSHM treatments compared with control. Based on these observations, WSHM was concluded to have positive effects on the nodulation of soybean inoculated with *B. liaoningense* CCBAU05525.

### The effects of WSHM on the Growth of *B. liaoningense* CCBAU05525

**Figure 1:**
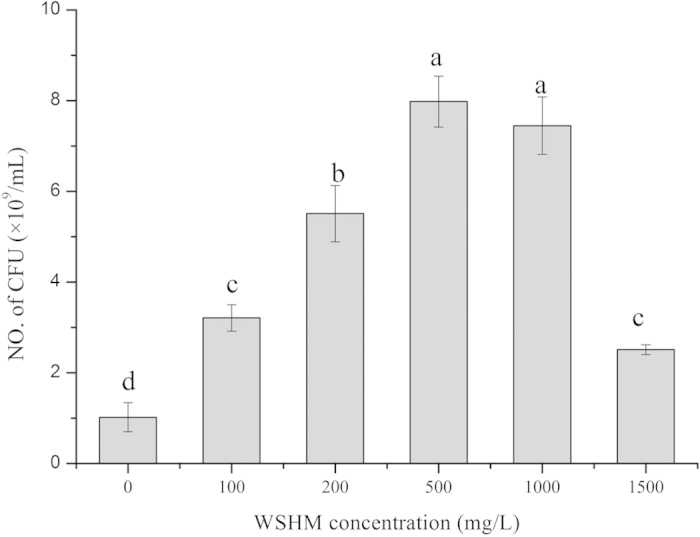
Effects of WSHM on the growth of *Bradyrhizobium liaoningense* CCBAU05525 in YM medium. The data were obtained on the fourth day of incubation with shaking 140 rpm at 28 °C. Values given are mean ± standard deviation of triplicate samples; Bars with different letters are significantly different (P < 0.05) from each other, according to LSD test.

As shown in Fig. 1, the cell density of *B. liaoningense* CCBAU05525 in YM broth was significantly increased by WSHM treatments at the concentrations of 100, 200, 500, 1000 and 1500 mg L^−1^. The highest cell density (7.98 ± 0.56 × 10^9^ cfu mL^−1^) on the fourth day of incubation observed at the WSHM concentrations of 500 mg L^−1^ was almost 8 times greater than that of control. The growth curves of *B. liaoningense* CCBAU05525 (available as Supplementary Fig. S1) showed that CCBAU05525 in the 500 mg L^−1^ WSHM solutions increased by 9.9 × 10^7^ mL^−1^. Whereas, the cell density of *B. liaoningense* CCBAU05525 in YM broth culture supplied with WSHM increased to 8 × 10^10^ mL^−1^, which was 8 times greater than that of *B. liaoningense* CCBAU05525 in YM broth culture without WSHM.

### Identification of the chemical compounds in WSHM

By TMAH-py-GC/MS analysis, 68 compounds were identified in WSHM which fell into 4 classes: aromatics, aliphatic, nitrogen compounds and other compounds (Table 2). The aromatics that included methoxy and hydroxy benzenes, aromatic ketones and phenolic acids were the main components of the WSHM. Fatty acid methyl esters and dicarboxylic acid dimethyl esters were detected in WSHM. Sterol isomers and diterpene resin acids contents, originated mainly from lipids were also one of the important components in WSHM. Besides, N-containing compounds such as pyrroles, imidazoles, pyrimidines, piperidines and indoles were identified. The indole-like compounds identified in WSHM, indicated its potential in promoting plant growth. In addition, it was noteworthy that the 3-Hydroxy-7-methoxy-3-phenyl-4-chromanone in the WSHM had similar structure of flavonoids (C6-C3-C6). Thus, WSHM might act as flavonoid in the symbiosis between soybean and rhizobia.

### Proteome analysis of *B. liaoningense* in response to WSHM and genistein

**Figure 2:**
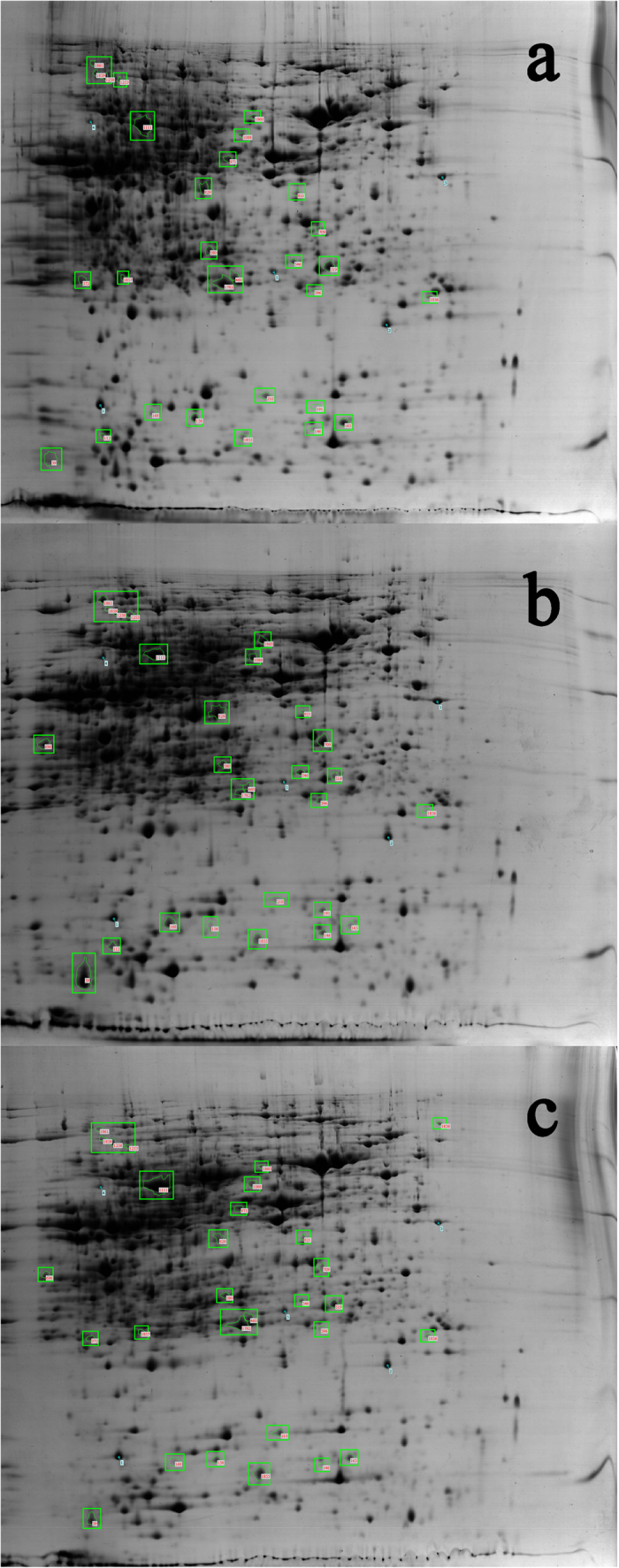
2-D Electrophoresis of the proteins of *B. liaoningense* CCBAU05525 following different treatments. a: control; b: treatment with water-soluble humic materials; c: treatment with genistein.

In the assay of proteomics, a total of 1,900 protein spots were identified by 2-D electrophoresis across all samples, 15 of which were up-regulated and 15 down-regulated by WSHM treatment. 7 and 9 proteins were up- and down-regulated respectively by genistein , all of which were among the 30 WSHM-affected proteins (Fig. 2). Among these 30 proteins, 27 were identified based on their similarity with reported sequences, which were divided into three groups: 1) protein expression was increased or decreased by both WSHM and genistein treatments; 2) protein expression was increased by genistein but decreased or not significantly altered by WSHM; 3) protein expression was increased by WSHM but decreased or not significantly altered by genistein (Table 3).

Proteins belonging to the first category included nitrogen regulatory protein PII, iron-regulated outer membrane protein, isopropylmalate isomerase small subunit, GroEL protein, replication protein A, and ATP synthase FliI/YscN, which were all up-regulated, and glutamine synthetase protein, DNA polymerase III subunit delta, DNA-directed RNA polymerase, beta subunit, aminoacyl-histidine dipeptidase, hypothetical protein CAMRE0001_2526, glycosyl hydrolase family 3 protein, DNA-cytosine methyltransferase, and Lon-A peptidase which were all down-regulated. Aldehyde dehydrogenase, sensor histidine kinase, hypothetical protein Swoo_4462, and the putative phosphoribulokinase protein which fell into the second category were all up-regulated by genistein but down-regulated by WSHM. Molybdenum ABC transporter, threonine ammonia-lyase, ACT domain-containing protein, hypothetical protein amb3581, putative phage-related protein, ABC transporter substrate-binding protein and hypothetical protein xccb100_1606 in the third category were all up-regulated by WSHM but down-regulated by genistein.

### QRT-PCR assay

Quantitative real-time PCR (QRT-PCR) showed that most gene expression data were consistent with the results of the analysis of proteomics, although differences in the rate of gene expression existed between the WSHM treatment and the control (Table 4). For example, nitrogen regulatory protein PII was up-regulated 5.58-fold by WSHM according to the proteomics results (Table 3), but a 17.26-fold difference was observed with RT-PCR (Table 4). Likewise, the apparent expression of the DNA-directed RNA polymerase beta subunit and the putative phosphoribulokinase protein differed significantly with the two methods. These differences might be a reflection of the post-transcriptional and/or post-translational phenomena and required further investigation.

**Figure 3:**
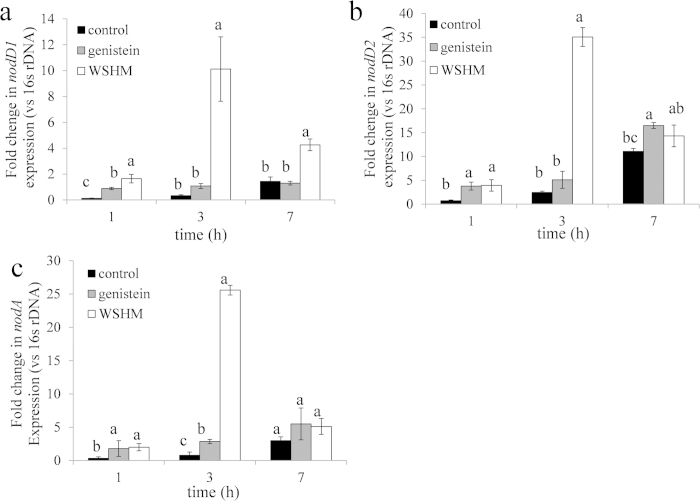
The expression pattern of *nodD* and *nodA* genes in *Bradyrhizobium liaoningense* CCBAU05525 induced by water-soluble humic materials or genistein. a: *nodD1*; b: *nodD2*; c: *nodA*. The data are expressed as mean ± SD values (n = 3). The statistical significance among the data set was assessed by LSD test (P < 0.05).

Besides, *nod* gene expression changes resulted from WSHM or genistein treatments were also investigated. QRT-PCR analyses revealed that the expression of *nodD*1, *nodD*2 and *nodA* increased significantly (P < 0.05) by WSHM treatment (Fig. 3). The expression of these genes reached the highest level 3 hours after the addition of WSHM to the cultures, which were 29.7-, 14.3- and 32.8-fold to those of the control respectively. However, the expression increase came to a halt, except for *nodD1*, 7 hours after WSHM addition. Similar results were observed in the genistein treatment, but the induction by genistein was significantly lower than that by WSHM.

## Discussion

Symbiotic nitrogen fixation plays an important role in sustainable agriculture, and this study demonstrated that WSHM, the humic acids, produced by biodegradation of lignite, possesses the ability to enhance symbiotic association between *B. liaoningense* CCBAU05525 and soybean plants. A WSHM concentration of 500 mg L^−1^ resulted in the maximal enhancement of cell density and nodulation of *B. liaoningense* CCBAU05525. According to our other studies, 375 g WSHM per hectare per year would contribute to 12.6% ~ 26.3% increases in the soybean yield (Our unpublished data). Furthermore, the identification of indole-like compounds in WSHM (Table 2) indicated the potential of WSHM in promoting plant growth. Since the WSHM used in this study was water, acid and alkali soluble. Thus, it was not only economically feasible but also profitable to use WSHM as a fertilizer in agriculture.

It was reported that humic acids could not only acting as C or energy source, but also stimulate bacterial growth by regulating cell metabolism^20^,^21^. In this study, similar results were observed. As shown in supplementary figure S1, the cell density of *B. liaoningense* CCBAU05525 in the 500 mg L^−1^ WSHM solution increased, which meant that WSHM could be used by the bacterium as nutrients; while the eight times of growth increase of CCBAU05525 in the YM broth supplied with 500 mg L^−1^ WSHM evidenced its stimulation function. Furthermore, the cell density in treatments of 1000 mg L^−1^ and 1500 mg L^−1^ WSHM were lower than that in 500 mg L^−1^ WSHM treatment (Fig. 1), indicating that 500 mg L^−1^ was the optimum concentration of WSHM in promoting growth of *B. liaoningense* CCBAU05525. Nonetheless, the 500 mg L^−1^ WSHM used in this study contained 52.18% and 3.72% of C and N, respectively^18^, which might account for the diauxic growth pattern of the bacteria in WSHM-treated cultures after the 7^th^ day (Supplementary Fig. S1). These results all suggested that WSHM could serve as nutrients in the growth of *B. liaoningense* CCBAU05525, but their nutrient effects were negligible. Thus, the stimulation effects of WSHM on *B. liaoningense* CCBAU05525 growth was mainly attributed to its regulation on cell metabolism and nutrient transport which were demonstrated in the proteomics assay.

In the proteomics assay, proteins of the first category might be involved in the early stages of nodulation, such as Nod factor biosynthesis or the switch from the free-living to the symbiotic living stage. The changes in expression levels (up or down) of these proteins remained the same in both WSHM and genistein treatments, but the WSHM treatment caused a consistent greater increase or decrease than the genistein treatment, indicating that the effects of WSHM were stronger than those of genistein. The differences observed in the second and third categories demonstrated that WSHM had different effects on *B. liaoningense* CCBAU05525 compared with genistein. Other chemical compounds in WSHM except for flavonoid analogue might also have positive effects on the nodulation of *B. liaoningense* CCBAU05525 and be even better than genistein.

Belonging to the first category, nitrogen regulatory protein PII was an essential component of a highly efficient system that regulated nitrogen assimilation. This system coordinated the intracellular concentration of glutamine and 2-ketoglutarate to ensure the appropriate regulation of glutamine synthetase (GS) activity and expression of the glnALG operon^22^,^23^. Besides, it was a fundamental step to switch off assimilating ammonia during bacteroid development. Furthermore, most aspects of cell growth and division, including the synthesis of nucleic acid were reduced during bacteroid development^2^, while, ATP synthesis was always essential to nodulation, protein synthesis and active transport. Considering the fact that glutamine synthetase protein, DNA polymerase III subunit delta, DNA-directed RNA polymerase beta subunit and DNA-cytosine methyltransferase were down-regulated and nitrogen regulatory protein PII and ATP synthase FliI/YscN protein were up-regulated by WSHM and genistein, the WSHM (like genistein) probably prepared the bacteria for better bacteroid development.

The chaperonin GroEL which played an essential role in ensuring proteins properly folded, preventing and repairing harmful effects, was highly conserved in evolution^24^,^25^. This protein enhances the expression of *nod* genes^26^, and was essential to the formation of a functional nitrogenase complex in the bacteroids of *B. japonicum*^27^. In addition, it interacted with the NifA polypeptide and enhanced *nif* gene expression^28^. Thus the upregulated expression of GroEL demonstrated that WSHM had positive effects on the expression of *nod, nif* genes and the formation of nitrogenase complex.

It was reported that molybdenum ABC transporter permease protein, molybdenum processing protein, molybdenum transport system permease protein and many ABC transporter substrate-binding proteins were only found to be expressed in 21-day-old bacteroids of *Bradyrhizobium japonicum* but not in free-living cells^29^. The molybdenum ABC transporter and iron-regulated outer membrane protein transported molybdenum and iron respectively. Iron and molybdenum were essential to nitrogen fixation because of their necessity as nitrogenase co-factors^2^. Therefore, up-regulating of these three transporter-associated proteins might be the reason for the increase in nodule fresh weight and nitrogenase activity of soybean by WSHM treatment.

With the proteomic assay, it was concluded that WSHM regulated the nitrogen metabolism, carbon metabolism, energy production, nutrient transport and signaling of *B. liaoningense* CCBAU05525. On one hand, WSHM had similar but superior effect on the expression of proteins in category 1 compared with genistein. On the other hand, WSHM had different effects on the expression of some proteins related to nitrogen fixation compared with genistein. Specifically, the protein expression pattern changed by WSHM was beneficial to the bacteroid development and the increase in nitrogenase activity.

Flavonoids secreted by host plant induce expression of *nodD* gene which then regulated the transcription of the structure genes (such as *nodABC*) to synthesis Nod factor^30^. Therefore, they were essential to the symbiosis between the legumes and rhizobia^31^. It was shown in this study that WSHM increased *nodD1, nodD2, nodA* gene expression of *B. liaoningense* CCBAU05525, which might be the reason for the increase in the nodule number and nitrogenase activity of soybean in the greenhouse experiments. The enhanced expression of *nod* genes by humic acids in *B. liaoningense* had not been reported previously. As expected, genistein also increased the expression of *nodD1, nodD2, nodA* gene. WSHM contained flavonoid analogue, which might account for its contribution to *nodD1, nodD2, nodA* gene expression. However, WSHM treatment caused a greater increase in the expression of these genes than genistein did, similar to the tendency of the proteins in category 1 shown in proteomics assay, indicating that the effects of WSHM were stronger than those of genistein. However, the higher concentration of WSHM used in the experiment might partially account for the greater effects. In addition, another explanation might be the complex mixture of components present in the WSHM treatment (Table 2), in which there might be some strong inducers for particular metabolic procedures; since *B. liaoningense* CCBAU05525 responded to WSHM and genistein differently in category 2 and 3 in proteomics assay. These strong inducers in the WSHM compounds had great potential in the development of new materials to stimulate the symbiotic nitrogen fixation between rhizobium and legume.

The failure to find NodD1, NodD2, and NodA in the analysis of proteomics might be due to their low abundance, since all of them are regulators for the expression of other genes. The function of several hypothetical proteins affected by WSHM treatment would be examined in future work. Also, the mechanism of WSHM in promoting nodulation of soybean with *B. liaoningense* CCBAU05525 needed to be further investigated, such as the effects of WSHM on the formation of infection thread of *B. liaoningense* CCBAU05525.

Conclusively, WSHM treatment increased the number of soybean nodules, the nodule fresh weight and the nitrogenase activity, as well as enhanced the cell density of *B. liaoningense* CCBAU05525. WSHM contained flavonoid analogues and showed similar bioactivities with genistein but had wider effects than genistein such as inducing the expression of *nodD1, nodD2, nodA*, and up-regulating a range of proteins involved in a variety of metabolic pathways. Furthermore, several nitrogen fixation-associated proteins such as molybdenum ABC transporter were also affected. Therefore, WSHM might prepare the free-living rhizobia for better bacteroid development. This was the first study about the effects of humic acids on rhizobia growth, *nod* gene expression and proteomics. These results shed new light on the effects of humic material on legumes and demonstrated great application value in improving soybean production.

## Materials and methods

### Water-soluble humic materials

Lignite was collected from the Huolingele Minerals Administration coal mine, Inner Mongolian Autonomous Region, Northwest China. Water-soluble humic materials (WSHM) was extracted according to a previously described protocol^32^.

### Plant nodulation test

*B. liaoningense* CCBAU05525 was obtained from the Culture Collection of China Agriculture University, Beijing, China^33^. This strain was cultured aerobically at 28 °C in YM broth (Mannitol, 10 g; NaCl, 0.1 g; K_2_HPO_4_, 0.25 g; KH_2_PO_4_, 0.25 g; Yeast Extract, 0.8 g; MgSO_4_·7 H_2_O, 0.2 g; pH 6.8–7.0). Bacterial culture with approximately 10^6^ cells and 5 mL of the WSHM solution with different concentration (0, 300, 500 and 1000 mg L^−1^) were used for inoculating soybean. Soybean seeds were surface-sterilized by successive treatments with 95% ethanol for 30 sec and 0.2% HgCl_2_ for 5 min, and were then washed for 6 times by sterile water. Then the surface-sterilized seeds were germinated on 0.8% agar-water plates in the dark at 28 °C for 24–48 h. Germinated seeds were planted in vermiculite moisturized with low-N nutrient solution in pots^34^ and were inoculated with 1 mL of bacterial culture and 5 mL of WSHM solution or 5 mL of sterile water as control per plant. For each treatment, 30 pots were selected and divided into three parallel groups. In each group, there were 10 plants in 10 pots. The average value of each group was taken as one sample value. Plants were grown in greenhouse at 25/17 ± 2 °C for day/night with 60% relative humidity. Pots were rearranged daily to give a random distribution of growth conditions in the greenhouse. After 35 days, all the soybean plants were harvested to detect the number, weight and nitrogenase activity (acetylene reduction assay) of the nodules^35^.

### Effects of WSHM on the growth of *B. liaoningense* CCBAU05525

It was known that the cell density of rhizobia around the plant roots must reach a threshold level for adequate nodulation. Thus, the effects of WSHM on the growth of *B. liaoningense* CCBAU05525 were analyzed. The strain was preincubated in 50 mL of YM broth for 4 days at 28 °C under shaking (140 rpm). Then, the CCBAU05525 culture was inoculated at a ratio of 1% (v/v) into 200 mL of YM broth supplied with WSHM at the final concentrations of 0, 100, 200, 500, 1000 and 1500 mg L^−1^, respectively. The flasks containing the inoculated YM broth were incubated at 28 °C under shaking (140 rpm) for four days up to the middle exponential phase and culture samples were taken to evaluate the rhizobial cell density by dilution-plating procedure. The results were then used to choose the suitable WSHM concentration for the study of the effects of WSHM on the growth curve (up to 10 days of incubation) and the *nod* gene expression and proteomics of rhizobia.

### TMAH-py-GC/MS of WSHM samples

The Py-GC/MS system used in this study was a combination of a PY-2020S pyrolyzer (Frontier Laboratories Ltd., Japan) and Shimadzu GCMS-QP2010 gas chromatograph mass spectrometer (Shimadzu, Milan, Italy) equipped with commercial mass spectral libraries (Nist107). All the WSHM samples (ca. 200 μg) were placed on a ferromagnetic pyrofoil, and 2 μL of tetramethyl ammonium hydroxide (TMAH) aqueous solution (10%, w/v) were added to the samples. The pyrolysis temperature was 550 °C, and the total heating time was 10 sec. The pyrolysis products were transported to the GC/MS, and separated on the GC equipped with a UA-5MS capillary column (Frontier Laboratories Ltd., Japan, 30 m × 0.25 mm ID, 0.25 μm thickness). The temperature protocol used was 6 °C min^−1^ from 40 °C (3 min) to 300 °C (10 min) with a heating rate of 10 °C min^−1^. The mass spectra of compounds were measured at 70 eV. The products released from pyrolysis were differentiated based on a search in the mass spectral library (Nist107) comparing the relative retention time and other Py-GC/MS data of humic acids (HA) and fulvic acids (FA)^36^,^37^.

### Protein preparation and extraction

*B. liaoningense* CCBAU05525 was pre-cultured aerobically at 28 °C to the early exponential phase (OD_600_ = 0.4 ~ 0.5) in 200 mL YM broth. Then, 100 μL of 1 mM (final concentration of 0.5 μM) genistein, which was a known flavonoid specific for the Nod factor induction in soybean rhizobia^38^,^39^; or 5 mL WSHM (at a concentration of 20 g L^−1^) were added and the incubation continued to the mid-exponential phase (OD_600_ = 0.7 ~ 0.8). Cultures without addition of the inducers were included as a control. Cells were harvested by centrifugation (8,000 × *g*, 10 min) at 4 °C, the cell pellet was washed with 50 mL NaCl solution (0.85%), resuspended in the same solution at a concentration of 0.5 g mL^−1^, and frozen at −80 °C in aliquots of 50–100 μL for Proteins extraction.

Proteins were extracted using the phenol/ammonium acetate method as described previously^40^ with slight modifications. Cell samples (1 g) were homogenized in 3 mL extraction buffer, and the protein concentration was determined using 2D Quant Kit (GE Healthcare) according to the instructions of the manufacturer.

### 2-D electrophoresis, imaging and data analysis

For 2-D electrophoresis, proteins were separated by isoelectric focusing (IEF) in the first dimension and SDS-PAGE in the second dimension. Samples were adjusted to a final concentration of 1.2 mg of protein in 450 μL DeStreak rehydration solution (GE Healthcare). Isoelectric point gel (IPG) strips (24 cm, diameter of 0.5 cm, pH 3–10; (GE Healthcare) were rehydrated with the sample and cover fluid and electrophoresed as described^41^. After IEF, the strip was equilibrated and placed on a large format 13% (w/v) polyacrylamide gel (26 × 20 cm) for SDS-PAGE as described^42^. After electrophoresis, the gel was stained with colloidal CBB (20% ethanol, 1.6% phosphoric acid, 8% ammonium sulfate, 0.08% CBB G-250) and imaged using a UMAX imagescanner. The image was analyzed using ImageMaster 2D Platinum v 5.0 software. At least three replicate gels of three different protein preparations were analyzed for consistency. If the change in the relative area of a protein spot was greater than 1.5-fold and shown to be statistically significant by the *t*-test (P < 0.05) using SPSS software, the protein expression level was considered to be modified by the treatment.

### In-gel digestion and protein identification

Protein spots that showed a significant difference (P < 0.05) in the 2-D electrophoresis experiment were excised manually, and in-gel digested overnight at 37 °C with sequencing grade modified trypsin (Promega, USA). Tryptic peptides (0.5 mL) were mixed with a saturated solution of α-Cyano-4-hydroxycinnamic acid (CHCA) in 50% (w/v) acetonitrile containing 0.1% trifluoroacetic acid. The mixture was spotted onto a MALDI sample plate and crystallized at room temperature. The same procedure was used for the standard peptide calibration mixture (Bruker Daltonics, Germany). Mass spectra were acquired using a MALDI-TOF-MS Autoflex spectrometer (Bruker Daltonics, Germany) operating in the PMF fully automated mode, or manually in the LIFT mode in the case of MALDI-TOF/TOF. PMFs and MS/MS ions were searched against the NCBI nr/Proteobacteria database using MASCOT software (Matrix Science, UK). The Kyoto Encyclopedia of Genes and Genomes (KEGG) database (http://www.genome.jp/kegg/) covering a total of 2270 *B. japonicum* genes and showing a complete or nearly complete pathway in several cases^43^ was used to identify the possible pathways affected by WSHM treatment. Enzymes and proteins annotated under the global KEGG category metabolism were identified.

### Quantitative Real-time PCR assay

Quantitative real-time PCR (QRT-PCR) was used to confirm the results of the proteomics experiments and detect the effects of WSHM on *nodD* and *nodA* gene expression. Cells were cultured as described above, except that the cells were collected at 1, 3 and 7 h after WSHM or genistein was added as inducer for *nod* gene expression. Total RNAs were isolated using TRIzol reagent (TIANGEN, Beijing) according to the manufacturer’s protocol. First strand cDNAs were synthesized using PrimeScript Reverse Transcriptase (RT) (TaKaRa Code: D2680S) according to the manufacturer’s instructions. RT samples were used for quantitative RT-PCR with the primers shown in Table 5, which were designed using the program Premier 5.0 based on the *B. liaoningense* CCBAU05525 genome sequence^33^. Expression of 16S rRNA was used as an internal control for normalization. Each reaction contained 10 μL of Power SYBR Green master Mix (ABI, USA) in a final volume of 20 μL. The PCR program was as follows: (i) 95 °C for 10 min, 40 cycles of 95 °C for 30 sec, 60 °C for 35 sec. PCR was performed on an ABI 7500 Thermo cycler and data were analyzed using ABI software (Foster City, USA).

## Additional Information

**How to cite this article**: Gao, T.G. *et al*. Nodulation Characterization and Proteomic Profiling of *Bradyrhizobium liaoningense* CCBAU05525 in Response to Water-Soluble Humic Materials. *Sci. Rep*. **5**, 10836; doi: 10.1038/srep10836 (2015).

## Supplementary Material

Supplementary Information

## Figures and Tables

**Table 1:** Effects of water-soluble humic material (WSHM) treatments on the nodulation of soybean in greenhouse^a^.

	WSHM concentration (mg L^−1^)			
Nodulation	0 (control)	300	500	1000
Number of nodules	36.0±1.00b	43.0±4.35a	47.0±3.00a	38.0±5.00b
Fresh weight of nodules (g)	0.436±0.0610b	0.547±0.0330ab	0.593±0.113a	0.554±0.0950ab
Nitrogenase activity (nmol C_2_H_4_ h^−1^)	22.0±2.93c	28.6±1.30a	25.3±0.320b	26.9±0.810ab

^a^Values are given as mean ± standard deviation of triplicate samples for nodule analysis; values with different alphabets in the same line are significantly (P < 0.05) different from each other, according to LSD test.

**Table 2:** Chemical structures in water-soluble humic material detected by TMAH-py-GC/MS.

Peak No.	Retention time (min)	Compound	Area%	Formula	Mr	CAS
1	2.300	2-methylfuran	0.69	C_5_H_6_O	82	534-22-5
2	2.395	Acrylic acid methyl ester	2.44	C_4_H_6_O_2_	86	96-33-3
3	2.535	Methyl propionate	1.87	C_4_H_8_O_2_	88	554-12-1
4	3.600	Methacrylic acid methyl ester	0.87	C_5_H_8_O_2_	100	80-62-6
5	4.210	2-chloroethyl methyl ether	2.25	C_3_H_7_ClO	94	627-42-9
6	4.415	(Dimethylamino) acetonitrile	4.43	C_4_H_8_N_2_	84	926-64-7
7	4.475	Pyrrole	2.45	C_4_H_5_N	67	109-97-7
8	4.710	Toluene	4.06	C_7_H_8_	92	108-88-3
9	5.145	2-Hydroxy-2-methyl-6-hepten-3-one	0.54	C_8_H_14_O_2_	142	996-61-2
10	5.575	3-(Octylamino) propanenitrile	0.58	C_11_H_22_N_2_	182	29504-89-0
11	5.690	Hexamethylcyclotrisiloxane	0.15	C_6_H_8_O_3_Si_3_	222	541-5-9
12	5.845	N,N-dimethyl-2-phosphinoethanamine	1.07	C_4_H_12_NP	105	161944-90-7
13	6.175	2-cyclopenten-1-one	0.82	C_5_H_6_O	82	930-30-3
14	6.755	Ethylbenzene	0.52	C_8_H_10_	106	100-41-4
15	6.815	2,5-Dimethylpyrrole	0.38	C_6_H_9_N	95	625-84-3
16	6.960	Dimethylbenzene	1.17	C_8_H_10_	106	95-47-6
17	7.435	1,3,5,7-Cyclooctatetraene	1.09	C_8_H_8_	108	629-20-9
18	7.960	Methoxybenzene	0.68	C_7_H_8_O	108	100-66-3
19	8.985	6-(Hydroxy-phenyl-methyl)-2,2-dimethyl-cyclohexanone	1.23	C_15_H_20_O_2_	232	CID557652 (NCBI no.)
20	9.205	Oktamethylcyklotetrasiloxan	0.39	C_8_H_24_O_4_Si_4_	296	556-67-2
21	9.555	Phenol	3.13	C_6_H_6_O	94	108-95-2
22	9.655	3-(Octylamino)Propanenitrile	1.03	C_11_H_22_N_2_	182	29504-89-0
23	10.095	(+)-dimethyl 2,3-O-benzylidene-D-tartrate	2.27	C_13_H_14_O_6_	266	38270-70-1
24	10.275	Butanedioic acid, dimethyl ester	2.86	C_6_H_10_O_4_	146	106-65-0
25	10.860	2-Methylphenol	0.77	C_7_H_8_O	108	95-48-7
26	11.010	2-Propenoic acid, 2-benzoylamino-3-phenyl,ethyl ester	0.57	C_18_H_17_NO_3_	295	32089-78-4
27	11.080	3-Ethyl-2-hydroxy-2-cyclopenten-1-one	0.35	C_7_H_10_O_2_	126	21835-1-8
28	11.350	3,4,4-Trimethyl-2-cyclopenten-1-one	3.25	C_8_H_12_O	124	30434-65-2
29	11.455	N-methylsuccinimide	4.4	C_5_H_7_NO_2_	113	1121-7-9
30	12.300	o-Dimethoxybenzene	0.94	C_8_H_10_O_2_	138	91-16-7
31	13.550	2,4-Imidazolidinedione,3,5,5-trimethyl-	0.8	C_6_H_10_N_2_O_2_	142	6345-19-3
32	13.600	S-(2,5-Dihydroxyphenyl) cyclohexylthiocarbamate	1.1	C_13_H_17_NO_3_S	267	345259-5-4
**33**	**14.465**	**N-methylindole**^a^	0.65	C_9_H_9_N	131	603-76-9
34	14.575	Dimethyl(2E,4E)-2,4-hexadienedioate	0.52	C_8_H_10_O_4_	170	1733-37-5
35	14.940	Benzeneacetonitrile	1.09	C_8_H_7_N	117	140-29-4
36	15.430	Piperidine, 1,1-methylenebis-	3.44	C_11_H_22_N_2_	182	880-9-1
37	15.855	1,2,4-Trimethoxybenzene	0.33	C_9_H_12_O_3_	168	135-77-3
**38**	**15.930**	**1,3-Dimethylindole**^a^	0.35	C_10_H_11_N	145	875-30-9
39	16.030	Benzoic acid, 4-methoxy-, methyl ester	0.5	C_9_H_10_O_3_	166	121-98-2
40	16.160	Methyl 5-oxo-2-pyrrolidinecarboxylate	2.55	C_6_H_9_NO_3_	143	54571-66-3
41	16.790	N-methylphthalimide	0.73	C_9_H_7_NO_2_	161	550-44-7
42	17.060	2,4(1H,3H)-Pyrimidinedione	2.01	C_7_H_10_N_2_O_2_	154	4401-71-2
43	17.440	3,4,5-Trimethoxybenzylamine	0.56	C_10_H_15_NO_3_	197	18638-99-8
44	17.955	Dodecanoic acid, methyl ester	1.370	C_13_H_26_O_2_	214	111-82-0
**45**	**18.485**	**(3Z)-1-Methyl-1H-indole-2,3-dione 3-hydrazone**^a^	0.490	C_9_H_9_N_3_O	175	3265-23-4
46	18.755	Methyl 3,5-dimethoxybenzoate	3.140	C_10_H_12_O_4_	196	2150-37-0
47	18.870	Benzoic acid, 3,4-dimethoxy-,methyl ester	0.920	C_10_H_12_O_4_	196	2150-38-1
48	20.425	3,4,5-Trimethoxybenzoic acid, methyl ester	1.950	C_11_H_14_O_5_	226	1916-7-0
49	20.480	Methyl tetradecanoate	0.910	C_15_H_30_O_2_	242	124-10-7
50	21.320	Pentadecanoic acid, methyl eater	0.510	C_16_H_32_O_2_	256	7132-64-1
51	22.115	1H-purine-2,6-dione, 3,7-dihydro-1,3,7-trimethyl	0.530	C_8_H_10_N_4_O_2_	194	58-8-2
52	22.350	Hexadecanoic acid, methyl ester	0.660	C_17_H_34_O_2_	270	112-39-0
53	22.760	Hexadecanoic acid, methyl ester	3.430	C_17_H_34_O_2_	270	112-39-0
54	23.510	4-[2-(4-Hydroxy-phenyl)-viny]-benzoic acid	0.850	C_15_H_12_O_3_	240	152027-61-7
55	23.680	Cyclopropaneoctanoic acid, 2-hexyl-,methyl ester	0.450	C_18_H_34_O_2_	282	10152-61-1
**56**	**23.745**	**3-Hydroxy-7-methoxy-3-phenyl-4-chromanone**^b^	1.140	C_16_H_14_O_4_	270	18380-57-9
57	24.590	9-Octadecenoic acid (Z)-,methyl ester	1.240	C_19_H_36_O_2_	296	112-62-9
58	24.645	9-Octadecenoic acid (Z)-,methyl ester	0.710	C_19_H_36_O_2_	296	112-62-9
59	24.830	Octadecanoic acid, methyl ester	1.340	C_19_H_38_O_2_	298	112-61-8
60	25.690	10-Nonadecenoic acid, methyl ester	0.460	C_20_H_38_O_2_	310	56599-83-8
61	26.085	2-Phenanthrenamine, 9,10-dihydro-7-nitro-	8.500	C_14_H_12_N_2_O_2_	240	18264-82-9
62	26.760	1-Phenanthrenecarboxylic acid, 7-ethenyl-1,2,3,4,4a,4b,5,6,7,8,10,10a -dodecahydro-1,4a,7-trimethyl-,methyl ester, (1R,4aR,4bS,7S,10aR)-	1.930	C_21_H_32_O_2_	316	1686-62-0
63	26.860	6-Methyl-4-phenyl-3-cyanopyridine-2(1H)-thione	1.980	C_13_H_10_N_2_S	226	78564-23-5
64	26.920	Methyl 5-[2-(3-furyl)ethyl]-1,4a-dimethyl-6-methylidene-decalin-1-carboxylate	2.070	C_21_H_30_O_3_	330	10267-15-9
65	27.060	Methyl dehydroabietyate	2.010	C_21_H_30_O_2_	314	1235-74-1
66	27.540	Methyl abietate	0.490	C_21_H_32_O_2_	316	127-25-3
67	29.145	7-Oxodehydroabietic acid, methyl ester	0.600	C_21_H_28_O_3_	328	110936-78-2
68	30.175	Tetracosanoic acid, methyl ester	0.440	C_25_H_50_O_2_	382	2442-49-1

^a^Indole-like Compounds.

^b^Flavonoid-like compound.

**Table 3:** Proteins identified in *B. liaoningense* CCBAU 05525 treated by genistein and water-soluble humic acid.

Category	Spot No.	Protein description*	Protein source	NCBI Access NO.	Participation in (function)	WSHM vs. control	Genistein vs. control
1	39	**nitrogen regulatory protein PII**	*Bradyrhizobium japonicum* USDA 110	gi|27375723	regulate glutamine synthetase	5.58	1.41
	168	**iron-regulated outer membrane protein**	*Mannheimia haemolytica* BOVINE	gi|261491674	adapt to the iron-restricted environment inside the host	3.35	1.15
	395	**isopropylmalate isomerase small subunit**	*Bradyrhizobium japonicum* USDA 110	gi|27375606	Leu biosynthetic pathway and the Met chain elongation	5.20	1.10
	695	**GroEL protein**	*Asaia bogorensis*	gi|269913095	proper folding of many proteins	3.38	2.31
	1252	**replication protein A**	*Agrobacterium radiobacter* K84	gi|222109068	DNA replication, repair and recombination	7.22	1.67
	1860	**ATP synthase FliI/YscN**	*Phaeobacter gallaeciensis* BS107	gi|163738066	ATP binding protein	13.17	2.76
	158	**glutamine synthetase protein**	*Rhizobium etli* CIAT 652	gi|190892623	degradation of amino acids	0.27	0.76
	165	**DNA polymerase III subunit delta**	*Rhodoferax ferrireducens* T118	gi|89899570	prokaryotic DNA replication	0.34	0.89
	209	**DNA-directed RNA polymerase, beta subunit**	*Neisseria polysaccharea* ATCC 43768	gi|297250922	transcription of DNA into RNA	0.46	0.97
	371	**aminoacyl-histidine dipeptidase**	*Vibrio mimicus* VM603	gi|258627332	catalyze dipeptides and tripeptides	0.20	0.97
	459	**hypothetical protein CAMRE0001_2526**	*Campylobacter rectus* RM3267	gi|223040483	Unknown	0.38	0.68
	518	**glycosyl hydrolase family 3 protein**	*Escherichia coli* MS 21-1	gi|300936554	Hydrolyse the glycosidic bond between carbohydrates	0.19	0.74
	579	**DNA-cytosine methyltransferase**	*Rhodopseudomonas palustris* DX-1	gi|316931670	transfer of methyl group to DNA	0.40	0.72
	820	**Lon-A peptidase**	*Novosphingobium aromaticivorans* DSM 12444	gi|87199382	regulates gene expression	0.20	0.55
	970	**hypothetical protein VMC_13940**	*Vibrio alginolyticus* 40B	gi|269965870	Unknown	0.25	0.91
	1781	**conserved hypothetical protein**	*Pseudovibrio* sp. JE062	gi|254470700	Unknown	0.02	0.88
2	1110	aldehyde dehydrogenase	*Bradyrhizobium japonicum* USDA 110	gi|27377927	oxidation of aldehydes	0.40	1.43
	1854	sensor histidine kinase	*Geobacter sulfurreducens* KN400	gi|298504392	signal transduction	0.65	3.78
	1856	hypothetical protein Swoo_4462	*Shewanella woodyi* ATCC 51908	gi|170728783	Unknown	ND	1.22
	1858	putative phosphoribulokinase protein	*Bradyrhizobium japonicum* USDA 110	gi|27377693	Carbon fixation etc.	0.67	1.95
3	112	threonine ammonia-lyase, biosynthetic	*Vibrio harveyi* HY01	gi|153834318	biosynthesis of amino acids	Unique	ND
	147	molybdenum ABC transporter, periplasmic molybdate-binding protein	*Sideroxydans lithotrophicus* ES-1	gi|291613363	high affinity molybdate uptake system	2.43	0.95
	708	ACT domain-containing protein	*Pantoea* sp. At-9b	gi|317053226	amino acid and purine synthesis	3.30	0.49
	828	hypothetical protein amb3581	*Magnetospirillum magneticum* AMB-1	gi|83312680	Unknown	2.81	0.56
	1087	putative phage-related protein	*Rhizobium etli* GR56	gi|218672483	Unknown	1.61	0.58
	1859	ABC transporter substrate-binding protein	*Bradyrhizobium japonicum* USDA 110	gi|27376323	translocation RNA and DNA etc. repair	3.29	0.31
	1861	hypothetical protein xccb100_1606	*Xanthomonas campestris* B100	gi|188991002	putative peptidase/protease	5.59	0.64

^*^The proteins marked in boldface letters are those up regulated or down regulated by both the WS and Genistein.

**Table 4:** Expression of some genes induced by WSHM detected by Real Time PCR.

		Expression* (×10^−2^) induced by		
Spot No.	Protein	Genistein	Control	WSHM
39	nitrogen regulatory protein PII	1.14 ± 0.0100b	0.220 ± 0.0600c	3.76 ± 0.160a
209	DNA-directed RNA polymerase, beta subunit	6.35 ± 0.0500b	3.10 ± 0.0300b	59.4 ± 3.11a
395	isopropylmalate isomerase small subunit	6.26 ± 0.0800b	1.52 ± 0.0600c	30.7 ± 2.22a
1858	putative phosphoribulokinase protein	86.4 ± 3.99a	20.4 ± 0.270c	58.7 ± 1.42b
1859	ABC transporter substrate-binding protein	1.95 ± 0.210b	1.74 ± 0.510b	39.8 ± 0.800a
1860	ATP synthase FliI/YscN	0.610 ± 0.0400b	0.250 ± 0.0200c	2.47 ± 0.460a

Assays were performed in triplicate, and the mean values and SDs are shown. Values in the same line having different letters are significantly different from each other according to LSD test (P < 0.05).

^*^Fold change in gene expression (vs 16s rDNA).

**Table 5:** Primers used in this study.

Name of primers	Primer sequence (5’-3’)	Corresponding gene	Tm (°C)
16S rRNA gene (341f)	CCTACGGGAGGCAGCAG	16S rRNA	58.1
16S rRNA gene (534r)	ATTACCGCGGCTGCTGG		58.6
39F	TCGGCGTTCACGGTCTCA	*glnK*	61.1
39R	TGGCGTCGATGGTCTTGT		57.2
209F	TCGGCGAACTCATGGAGAA	Gene for DNA-directed RNA polymerase, beta subunit	60.5
209R	GCTGCGAGGAACCGAAGAA		61.1
1859F	CTCTACGAAGGCACCGACTG	Gene for ABC transporter substrate-binding	60.0
1859R	CGGGAATGACCTGCCAGTAG		60.3
395F	CGATCCGCGTCAGCCAGGAA	*leuD*	63.9
395R	AGGCAGTGCTTGCGGAACG		61.8
1858F	ATTCGGACCTGCTGTTCTACG	*cbbP*	59.4
1858R	TCCGCCGCAGAATGGTGT		62.7
1860F	GCTCGCGGTCGCCGAATAT	Gene for ATP synthase FliI/YscN	65.6
1860R	CGGCAACTCGGTGAAGACG		62.1
nodAF	GAGCCTTCGTTGGGAAAGTG	Gene for acyltransferase	60
nodAR	CGCAGTAGCCCGATGTGAG		60
nodD1F	CGCACTGAGACCGAACCT	Gene for a member of the NodD family of LysR-type transcriptional regulators	60
nodD1R	CAGTCGTAAGGGCATCGT		60
nodD2F	TTGGGACGAACCGCATAG	Gene for a regulator of Nod factor production	60
nodD2R	GGGTCGCTGTTGTGAAGTG		60
