# Supplementary material for: Nodulation Characterization and Proteomic Profiling of Bradyrhizobium liaoningense CCBAU05525 in Response to Water-Soluble Humic Materials
Source: Sci Rep. 2015 Jun 8;5:10836. doi: 10.1038/srep10836 (PMC4650689; doi:10.1038/srep10836)
Supplement: Supplementary Information [file srep10836-s1.pdf]

Nodulation Characterization and Proteomic Profiling of *Bradyrhizobium liaoningense* CCBAU05525 in Response to Water-Soluble Humic Materials

Tong Guo Gao[ab,1], Yuan Yuan Xu[a,1], Feng Jiang[a], Bao Zhen Li[a], Jin Shui Yang[a], En Tao Wang[c], Hong Li Yuan[a*]

a. State Key Laboratory of Agro-Biotechnology and MOA Key Laboratory of Soil Microbiology, College of Biological Sciences, China Agricultural University, Beijing 100193, P. R. China

b. College of Life Science, Agricultural University of Hebei, Baoding 071001, P. R. China

c. Escuela Nacional de Ciencias Biológicas, Instituto Politécnico Nacional, México D.F. 11340, Mexico

[*]Corresponding author: E-mail address: hlyuan@cau.edu.cn (H. L. Yuan)

[1]Co-first authors who contributed equally to the work.

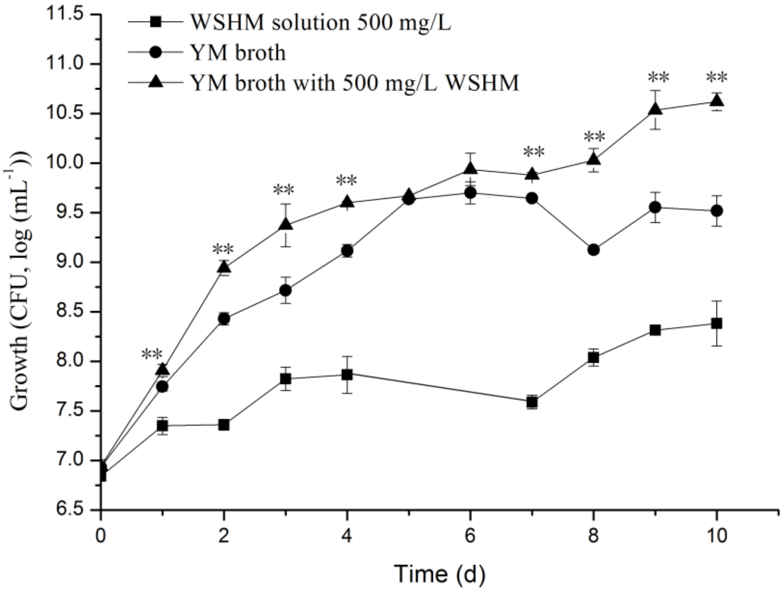

Supplementary Fig. S1. The effects of water-soluble humic materials on the growth of *B. liaoningense* CCBAU05525. The data are expressed as mean±SD values (n=3). Square: the growth curve of CCBAU05525 in WSHM solution with the concentration of 500 mg mL^{-1}. Circle: the growth curve of CCBAU05525 in YM broth. Triangle: the growth curve of CCBAU05525 in YM broth supplied with 500 mg mL^{-1} WSHM. The cultures were incubated at 28 ℃ with shaking (140 rpm). The statistical significance among the data set was assessed by LSD test (*P<0.05).
